# A citizen science based survey method for estimating the density of urban carnivores

**DOI:** 10.1371/journal.pone.0197445

**Published:** 2018-05-22

**Authors:** Dawn M. Scott, Rowenna Baker, Naomi Charman, Heidi Karlsson, Richard W. Yarnell, Aileen C. Mill, Graham C. Smith, Bryony A. Tolhurst

**Affiliations:** 1 Conservation Ecology and Zoonosis Research Group, University of Brighton, Brighton, United Kingdom; 2 School of Animal, Rural and Environmental Sciences, Nottingham Trent University, Brackenhurst Campus, Southwell, United Kingdom; 3 Centre for Wildlife Management, Newcastle University, Newcastle upon Tyne, United Kingdom; 4 Animal and Plant Health Agency, Sand Hutton, York, United Kingdom; Centre for Cellular and Molecular Biology, INDIA

## Abstract

Globally there are many examples of synanthropic carnivores exploiting growth in urbanisation. As carnivores can come into conflict with humans and are potential vectors of zoonotic disease, assessing densities in suburban areas and identifying factors that influence them are necessary to aid management and mitigation. However, fragmented, privately owned land restricts the use of conventional carnivore surveying techniques in these areas, requiring development of novel methods. We present a method that combines questionnaire distribution to residents with field surveys and GIS, to determine relative density of two urban carnivores in England, Great Britain. We determined the density of: red fox (*Vulpes vulpes*) social groups in 14, approximately 1km^2^ suburban areas in 8 different towns and cities; and Eurasian badger (*Meles meles*) social groups in three suburban areas of one city. Average relative fox group density (FGD) was 3.72 km^-2^, which was double the estimates for cities with resident foxes in the 1980’s. Density was comparable to an alternative estimate derived from trapping and GPS-tracking, indicating the validity of the method. However, FGD did not correlate with a national dataset based on fox sightings, indicating unreliability of the national data to determine actual densities or to extrapolate a national population estimate. Using species-specific clustering units that reflect social organisation, the method was additionally applied to suburban badgers to derive relative badger group density (BGD) for one city (Brighton, 2.41 km^-2^). We demonstrate that citizen science approaches can effectively obtain data to assess suburban carnivore density, however publicly derived national data sets need to be locally validated before extrapolations can be undertaken. The method we present for assessing densities of foxes and badgers in British towns and cities is also adaptable to other urban carnivores elsewhere. However this transferability is contingent on species traits meeting particular criteria, and on resident responsiveness.

## Introduction

Urbanization is a major cause of land use change worldwide, typically resulting in the loss of biodiversity [1]. Conversely, several behaviourally flexible mammalian carnivores have successfully exploited the ecological opportunities associated with urban development, becoming synanthropic such that their densities in urban habitat typically exceed those in natural landscapes [2, 3, 4]. In Europe examples of synanthropic species include the red fox (*Vulpes vulpes)* and Eurasian badger (*Meles meles*) [2]. Carnivore adaptations to urban environments include ecological and behavioural changes [5], which combined with increasing density can affect the type and magnitude of intra- and inter- specific interactions. Although urban wildlife can provide benefits to human residents [6, 7] the occurrence of high density carnivore populations in close proximity to humans raises concerns about potential conflicts [8, 9] including increased risk of zoonotic disease transmission [8, 10]. Therefore assessing urban carnivore density, understanding those factors which influence density, and determining patterns of temporal change are essential for devising strategies for management, mitigating conflicts and controlling zoonotic disease [11, 3].

Globally, the red fox is the most widespread and successful urban carnivore, having colonized cities in Europe, Australia, USA, Canada and Japan [12, 13, 14]. In Europe, it is host to a range of diseases and parasites including the zoonotic tapeworm *Echinococcus multilocularis* and rabies (*Lyssavirus* sp.) [15, 16]. Although foxes in Britain are currently free of *E*. *multilocularis*, this causative agent of human alveolar echinococcosis (HAE) is becoming more widely distributed in Europe, and continued surveillance is recommended [17]. Likewise, it is possible that rabies could be re- introduced to Great Britain and the occurrence of both red foxes and Eurasian badgers at high density has the potential to sustain a major epizootic [18]. Effective control of such an outbreak would require accurate estimation of urban fox and badger social group densities (hereafter termed FGD and BGD respectively) [18]. Rabies contingency plans for Britain developed in the 1980s were reliant on the relationship between FGD and human sociological data demonstrated at the time [19, 20]. However, in response to a growing human population, urban areas have expanded and urban landscape structure changed [21], as has the socioeconomic profile of residents and their interactions with wildlife [8, 7]. Simultaneously, urban fox abundance and distribution has altered [22, 23, 24, 25] and consequently there is a need to assess current FGD. Urban badgers are less widespread globally but increasing reports in Britain [2] concurrent with incidences of conflict [9] highlight the need to also assess badger density.

In general, foxes and badgers are most frequently found in residential, suburban areas [26, 5] occurring less frequently in city centers and industrial zones [27, 28]. However the patchwork of privately owned land in suburban areas restricts the use of conventional carnivore surveying techniques such as direct sightings from spotlighting, or indirect quantification of field signs [29]. However, the presence of residents lends itself to the use of questionnaire surveys and “citizen science” based approaches which have been used successfully to monitor urban mammals [30, 8].

Both foxes and badgers typically live in social groups that defend exclusive territories and seasonally produce one litter per year [31, 13]. As social group size varies considerably in both species, density is typically expressed as social group density. Harris [32, 33] devised a survey technique to determine urban FGD based on sightings by schoolchildren of litters of cubs, to determine the number of social units. Additionally, mean territory size was estimated from local radio-tracking and mean group size from capture-mark recapture studies. This method was subsequently used in several urban areas to derive data for populating predictive models to determine FGD in multiple cities in Britain [27]. However, feasibility of using school children as assessors varies locally and the expertise and expense required to trap and radio-track animals limits the method’s wider applicability [29]. Cub sightings to determine fox social groups and density have also been applied outside of Britain, for example in Melbourne, Australia [12].

Badgers typically use one main sett, plus annex setts and outlying holes [31]. A positive association between number of main setts and number of social groups forms the basis of badger social group density estimates in rural Britain [34]. Main sett counts combined with radio-tracking have been used to determine urban densities locally [5]. However wider transferability of main sett counts to estimate BGD within urban areas is challenging as the size and spatial configuration of urban badger group territories differ from rural ones [5, 35]. Therefore, an independent method of delineating urban social groups would be beneficial. Ideally, the method would be low-cost, implementable within suburban constraints and adaptable to other suburban carnivores and localities, potentially leading to rapid multi-carnivore assessments.

We used a combined questionnaire, field survey and GIS-based method to survey urban foxes and badgers. We aimed to: (i) determine and contrast current relative FGD estimates in suburban areas of selected towns/cities in England; (ii) compare these with historical estimates to determine any temporal changes; (iii) investigate if suburban landscape composition predicts FGD; and (iv) determine whether FGD correlates with relative sightings density of foxes across Britain from a previous dataset [24]. Furthermore, we explore the potential application of the method to another urban carnivore (Eurasian badger). Finally, we discuss the method’s wider applicability to other urban carnivores.

## Materials and methods

### Study sites

Urban fox sightings were previously collected from members of the public during April and May 2012 and used to calculate fox sightings density (FSD: the number of sightings per 1000 people km^-2^) for 144 towns/cities across Great Britain (for full methodology see [24]). However Scott et al. (2014) state that follow up on the ground field studies in a sub-set of cities should be conducted to quantify the relationship between ‘true’ densities relative to sightings density. Based on the FSD values, eight towns/cities in England deemed representative of FGD range in British cities were selected for the current study. Because of an historic north-south divide in urban fox distribution [36] four towns/cities were selected from northern and eastern England (Newcastle, Huddersfield, Preston, and Norwich) and four from the south (London, Bournemouth, Portsmouth and Brighton). To investigate variable FGD within suburban areas, more than one site was surveyed in four cities where such replication was logistically feasible, according to FSD categories (low-high) as follows: Huddersfield (N = 2 sites; low, FSD = 14.6), Brighton (N = 4; medium FSD = 37.8), Newcastle (N = 2; medium FSD = 51.2) and London (N = 2; high FSD = 703.1). All other cities were N = 1 due to survey logistics. In parallel with fox data collection, badgers were also surveyed in one city (Brighton, N = 3) that had known occurrence of badgers and a historical density estimate [5]. Blocks of approximately 1km^2^ were randomly selected at each site within each town/city. This size was deemed large enough for potential occupation by multiple social groups, but logistically feasible to survey within a narrow temporal window.

### Questionnaires and field surveys

Surveys were conducted between July and mid-August in 2013, 2014 and/or 2015. Questionnaires were distributed by hand to all residential buildings within each of the study sites, excluding those stating “no junk mail” (<0.1% of houses surveyed). Schools, hospitals, businesses and other buildings also received questionnaires to cover their grounds. To increase return rates, questionnaires included either a pre-paid return envelope or an institute-specific email address. All questionnaires additionally included an identical online survey link. In Huddersfield and Newcastle, questionnaires were combined with door-to-door enquiries to increase return rates. Questionnaires requested the respondent’s address, and asked whether they had sighted any fox cubs on their residence between April and July in the current and previous year. At three sites in Brighton we additionally asked residents to report badger sightings in their gardens and surrounding areas in the current and previous year and the location of any known badger setts in the area. Questionnaire data were submitted anonymously and voluntarily. Concurrently we undertook an active search of all the public green spaces and accessible unpaved ground within the 14 sites to locate any active fox earths. In Brighton, badger setts were also surveyed and sett type (main or other) recorded.

### Home range, buffer size and alternative estimates

Fox cubs typically emerge from their natal den aged 4–6 weeks (mid-April to May). Up until 12–14 weeks old (July) the area over which they range gradually increases to include a series of secure locations (“rendezvous sites”) typically spaced <200m apart [37]. Where territories encompass multiple litters, these are often pooled such that individual cubs with different mothers will be present at the same rendezvous sites [37, 38]. One consequence of this behaviour is that all the cubs present within one social group may be observed by multiple householders at multiple sites in any given year. A badger social group typically uses one main sett with annex setts and outlier holes, although when studying urban badgers in Brighton, Davison et al., (2008) found that annex holes were usually within 150m of the main sett. Consequently, for both species, it was necessary to devise a methodology for merging multiple records into a single cluster representing a social group.

To inform an appropriate buffer size for merging records, the mean 95% home ranges of urban foxes were determined from parallel GPS-tracking studies in Brighton [39]. Between 2012 and 2015, the authors tracked 20 foxes (12 males; 8 females) from different social groups and derived kernel density estimate (KDE) home ranges using at least 200 relocations per animal (for full field and computational methodology see [39]). Between 2014 and 2015, 6 badgers were trapped and GPS-tracked in Brighton (H. Karlsson, unpublished data) following a modified fox-trapping protocol in accordance with Natural England (NE) Guidelines. Procedures were conducted under Home Office licence PPL 7007429, and NE licence 2014-3671-SCI-SCI. All protocols were approved by the ethics committee of the School of Pharmacy and Biomolecular Sciences, University of Brighton.

A 200m buffer distance was selected for both species for merging observations based on: mean radial distances of 95% KDE home ranges (foxes = 14.2ha [±3.26 SE]; badgers = 6.53ha [±1.91 SE] (H. Karlsson, unpublished data); mean distance moved by fox cubs between rendezvous sites in July [37]; mean distance between main and annex setts within an urban badger social group; and main sett dispersion of <570m [35]. To validate FGD estimates, an additional density calculation method from field data was employed at the Elm Grove site (Brighton). GPS-tracking of neighbouring fox social groups was used to determine number of group territories, and territory size and configuration (see [39] for methods of live trapping and GPS tracking). During live trapping foxes were individually identified using external features and face markings. In addition camera traps (Bushnell, Kansas, USA) were placed at two locations at least 100m apart within each home range and operated for a minimum of seven nights. Group size was based on the minimum number of fox individuals recorded within the home range from both live trapping and camera trapping.

### GIS analysis

Questionnaires were checked for errors and any that were incomplete or obviously erroneous excluded. All questionnaires with valid addresses and reports of fox cubs, badger sightings or badger setts within the study area were spatially referenced using Ordnance Survey (OS) grid reference finder (http://gridreferencefinder.com). To accurately record the area that had been surveyed, all reported locations were plotted and a 50m buffer created around each return. Buffer size was based on local field experience: we considered 50m (equivalent to approximately two gardens in an area of semi-detached housing) a suitable distance within which a householder was likely to be aware of fox cub or badger sett activity. However, this buffering approach created a concave outline with cavities that were partially surrounded by gardens from which data were available. In the case of foxes, which occupy convex territories [26], such “pockets” would be likely to constitute part of the territory hence excluding them would result in underestimating the area surveyed, and over-estimating FGD. Consequently, concavities were integrated into the survey area in cases where at least 75% of the surrounding edge was covered by survey respondents and/or surveyed greenspaces from field data validation.

**Fig 1:**
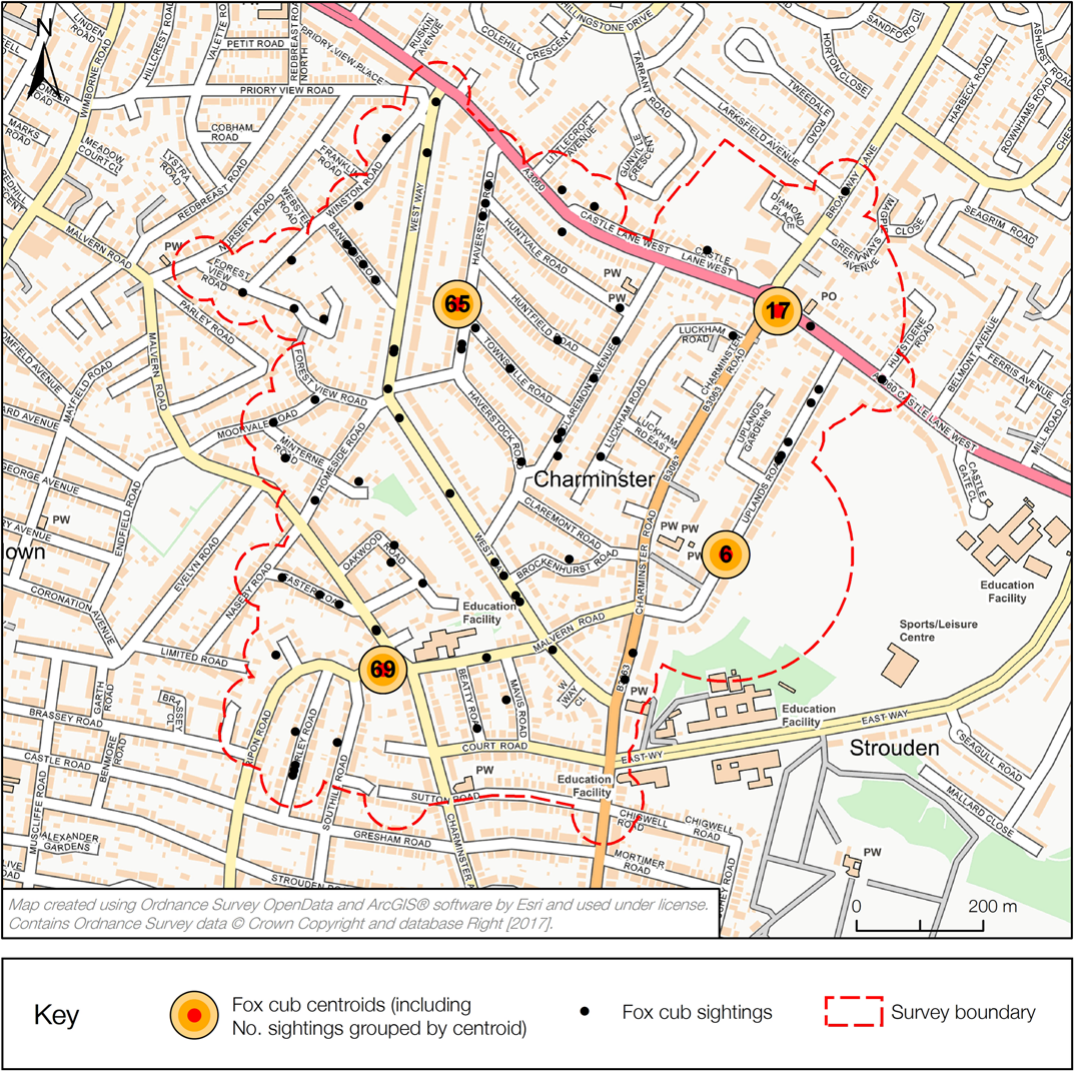
Fox cub centroid integration. An example of the integration method to derive the number of independent fox social groups from cub sightings for one survey site.

All fox cub records, badger sightings and setts spaced less than 200m apart were integrated into a single centroid point using the Integrate tool within ArcGIS (v 10.3.1). An additional 200m buffer area was then added around these points, and merged with the survey boundary to produce an overall survey area. Size of survey area (km^2^) was calculated using the ArcGIS Calculate Geometry tool and the number of independent centroids summed to estimate number of social groups within the survey area (See Fig 1).

Temporal changes in suburban fox occupancy were measured in terms of time since colonisation for each town/city: either ‘recent’ if resident foxes were present after 1986 only; or ‘long-term’ if present before 1986 [19].

England covers an area of 130,279 km^2^, of which 9% (11,690 km^2^) is classified as ‘urban’ [40] and 7% (9,116.4 km^2^) ‘suburban’. Suburban areas contain 55% of the human population in England and Wales [39]. To determine whether suburban landscape structure influenced FGD, housing density and the spatial extent (area) of green space, residential gardens, industrial area and manmade surfaces were calculated per km^2^ within the survey area from Ordnance Survey master map topography (https://www.ordnancesurvey.co.uk/business-and-government/products/topography-layer.html). Suburban landscape variables used in the study are described in Table 1, listing features included and excluded and the spatial statistics derived from each survey area. The spatial extent of each land use type was validated using satellite imagery (Bing maps and Google Earth).

**Table 1:** Land use types, features included and excluded and spatial statistics.

Land use type	Inclusions	Exclusions	Spatial Statistic
Residential Dwellings	• Houses • Flats • Care homes • Halls of residence	• Sheds and garages • Churches • Industrial buildings • Train stations • Shopping centres • Incomplete dwellings resulting from clipping OSMM to survey boundary	Housing density No. houses km^-2^ (HD).
Residential Gardens	• Gardens of residential dwellings • Walled areas around flats	• Church yards • School grounds • Incomplete gardens resulting from clipping OSMM to survey boundary	Garden area km^-2^ (GA)
Urban Green Space	• Amenity grassland/parks • Extensive road verges/islands • Church yards • School grounds • Allotments • Sports grounds • Railway embankments	• Hard ground fenced tennis courts	Urban green space km^-2^ (GS)
Made Ground	• Manmade Surfaces: • Roads and verges • Car parks • Tennis courts • School playgrounds	• Any natural environment within these	Made ground km^-2^ (MG)

Land use types with features included and excluded per category and spatial statistics derived from each survey area. OSMM–Ordnance Survey Master Map. Any additional areas or exclusions were identified using online satellite imagery.

### Statistical analysis

To determine whether FSD from Scott et al.’s (2014) national fox sightings study could constitute a proxy for FGD and thus allow extrapolation to other cities using estimates from comparable urban areas, we explored the relationship between these two non-normally distributed variables using Spearman’s rank correlation. To identify landscape variables potentially predicting FGD, we scrutinized five continuous variables (green space [GS], garden area [GA], housing density [HD], industrial area [IND] and made ground [MG]), and one binary variable (time since colonisation [COLON]: recent or long-term). FGD was square-root transformed to stabilise variances and consequently modelled with a Gaussian error distribution and a log link function. Collinearity between predictors was checked post- analysis using a Variation Inflation Factor (VIF) procedure, where VIF coefficients were calculated for each variable, and the largest value removed sequentially until all remaining values were within acceptable bounds (i.e. VIF <2.5). This resulted in the removal of garden area as a candidate model term (VIF = 45.67). As multiple densities were calculated for some towns/cities, we included CITY, with 8 levels, as a random factor in our Generalised Linear Mixed Model (GLMM). A backwards stepwise model selection procedure was employed where all fixed effects were initially entered together before sequential removal of non-significant variables at the 95% level, based on lowest deviance values. All statistical analyses were computed in R 3.4.0 [40] and the GLMM was computed using the glmmPQL command in package MASS.

## Results

Of ~30,000 questionnaires distributed across a total survey area of 17km^2^ across eight towns/cities, 5645 responses (19%) were received; an average response of 403 questionnaires per study area (n = 14, SE = 61.3). This resulted in 1802 fox cub sightings. Field studies verified locations reported from the questionnaires but did not add additional cub sightings to the data. Using cub sightings integrated at 200m, mean FGD was 3.63 km^-2^ (95% CI of mean: 3.11–4.59) (Table 2). The alternative density estimation method used in 0.72km^2^ of Elm Grove (Brighton) gave a FGD of 4.17 km^-2^ and an individual fox density of 13.9 foxes km^-2^ if multiplied by the local mean group size of 3.33 adults (n = 3). This was broadly similar but slightly higher than that derived using cub sightings integrations in a nearby, and partially overlapping location (FGD = 3.39 km^-2^, individual density = 11.5 km^-2^ if multiplied by average group size 3.4 following [41]). Precision of replicates was relatively high in Brighton and Huddersfield but lower in Newcastle and London (Table 2). Of the seven towns/cities with historical estimates for fox social groups [36] four that had previously not supported any established groups now did so, and the remaining three showed an increased FGD. The magnitude of this increase varied. In cities which had previous established fox populations (London, Brighton and Portsmouth) the average FGD had doubled from 1.73 to 3.51 km^-2^. In six areas that had not previously supported any groups, current mean FGD was 3.71 km^-2^. If densities in these recently colonised cities were included, the average FGD overall was five times that of previous estimates. If average FGD across cities (3.72) was multiplied by total suburban land cover this would equate to 33,092 [± 2,279 SE] fox social groups in suburban England, and a mean of one fox group per 648 houses across our study areas.

**Table 2:** Fox and badger group density and landscape composition of study sites.

City	Region	Area surveyed (km^2^)	No. of response	Green space(km^-2^)	Garden area(km^-2^)	Housing density(km^-2^)	No. of fox cub records	FSD	FGD(km^-2^)^(previous)^	Average FGD/ city (km^-2^) (SE)	BGD(km^-2^)	Houses/fox family group
London^1^	Croydon	1.04	77	0.168	0.515	1093	45	703.1	3.84 (0.7±0.4)	3.11 (1.02)		285
	Surbiton	0.84	240	0.196	0.511	1614	232		2.39			675
Brighton^1^	Elm Grove	1.18	752	0.126	0.281	4266	129	37.8	3.39 (2.0±0.2)	3.53 (0.53)	3.70	1260
	Hove	1.43	306	0.138	0.558	1155	120		3.49		2.67	331
	Portslade	1.18	369	0.175	0.433	2149	137		4.25		0.87	506
	Preston park	1.68	459	0.391	0.350	1104	256		2.98			370
Bournemouth^1^		0.92	366	0.085	0.536	1986	159	72.4	4.35	4.35		457
Portsmouth^1^		0.51	138	0.010	0.318	6028	21	13.2	3.91 (2.5±0.3)	3.91		1540
Newcastle^2^	Fenham	4.16	607	0.293	0.276	1907	38	51.2	1.68 (0)	3.24 (2.21)		1133
	Heaton	0.83	395	0.244	0.389	1770	48		4.81			368
Huddersfield^2^	Almondbury	1.06	568	0.397	0.320	1530	337	14.6	4.73 (0)	4.15 (0.82)		324
	Lockwood	0.84	858	0.262	0.196	2118	253		3.57			593
Norwich^2^		0.63	147	0.075	0.577	2087	19	36.6	4.73 (0)	4.73		441
Preston^2^		0.72	363	0.138	0.528	2177	8	4.0	2.76 (0)	2.76		789
Total		17.02	5645				1802					
Mean				0.193	0.413	2213			3.63 (0.74)	3.72	2.41	648
SE				0.03	0.03	360			0.25 (0.40)	0.24	0.83	394

Relative fox group density (FGD) and badger group density (BGD) estimates and landscape composition in the 14 study sites.

^1^cities are considered long established

^2^ cities with recent colonisation

No. of response = number of respondents data used in analysis, No. of fox cub records = number of fox cub records in one site over 2 years. FSD = Fox sightings density from [24]; ^(previous)^ = values in parenthesis denote predicted fox social group densities from [36].

Of the 6000 questionnaires distributed across three sites in Brighton, 1426 respondents reported 167 badger setts or sightings. Field studies verified and added locations and types of setts in the surrounding area to add to the distribution maps prior to analysis. This resulted in a mean BGD of 2.41 [±0.83 SE] and a mean individual density of 13.3 [± 4.56 SE], using 5.5 as group size (Table 2). If this value was representative of urban badger density, multiplication by suburban land cover would give an estimate of 21,971 [± 7,567 SE] badger social groups in suburban areas in England.

Fox sightings data from Scott et al. (2014) were not correlated with FGD (Spearman’s rho, r_s_ = 0.048, df = 7, P = 0.935). None of the landscape variables nor time since colonisation (COLON) predicted FGD (GLMM, full model deviance divided by null model deviance = 6.3%, *t* always < 0.9, P always > 0.05) and CITY accounted for only 0.003% of the residual variance (0.0001/3.79).

## Discussion

Judge et al, 2014 [34] state that social group abundance may be considered as useful as total population size (i.e. number of individuals) as it is less likely to vary at fine temporal scales, and is epidemiologically informative for disease transmission in social carnivores. Additionally, management actions are typically taken at group level. For both foxes and badgers, social group density estimates allow data to be compared historically and between different studies, whilst maintaining relevance to disease models [18]. Where an estimate of total population size is required, future studies additionally need to determine accurate current mean group size for their target species. In this study, we extrapolate from fox group density to individual (total population) density by multiplying up with a group size of 3.4 (as used in [41]. Our field validation exercise yielded a comparable group size of 3.3. However, fox group size is documented to be highly variable, with up to 10 adults in a group in Bristol at peak population density [22]. Furthermore, a positive association between group density and group size cannot be assumed; for example, historical control operations resulted in a reduction in the number of individuals per group, but not the number of groups [36]. Likewise, long-term studies of rural badgers have shown temporal stability in social group density despite overall increases in population size [42] indicating that group size, rather than number of groups, was the unit of change. We used a group size of 5.5 to estimate individual density of badgers [35], however badger group sizes can also vary widely and are constantly in flux due to mortality and dispersal [31].

In this study, we recalculate fox group density in multiple cities in Britain for the first time since the 1980s. Our findings show similar group densities across towns/cities sampled between 2013 and 2015. However, we report marked increases since the 1987 study [36]. Our protocol is based on the same fundamental principles and therefore we have assumed direct comparability, although we recognise that both survey approaches may contain inherent errors and caution must be used when inferring differences/similarities. With this caveat in mind, we found the number of fox social groups in towns/cities with previously established fox populations to be double that of Harris & Smith [36], and foxes to be present in all previously un-colonised towns/cities. As fox density varies within and between cities, we cannot assume that this magnitude of change has occurred across Britain, although an overall increase appears plausible given the increased number of urban areas with foxes [24]. We found social group densities to range from 1.68 to 4.81 km^-2^, the higher limit of which exceeds that previously used to develop rabies control models (maximum density 4 groups km^-2^ [11]). It would therefore be advisable to revisit these models with the view that fox densities are now likely to be higher in many cities. As the method was based on fundamental principle of independent natal dens, FGD is also comparable with other countries, for example FGD in Melbourne, Australia, was estimated to be between 0.47and 2.55 km^-2^ [12].

Behavioural adaptation to exploit urban habitats, combined with natal habitat preference [43], suggest that foxes born and raised in urban areas are more likely to remain there, contributing to the maintenance and expansion of populations [44]. Once colonisation has occurred, population growth will be determined by resources available e.g. food, rest and den sites [22] and by mortality and emigration. Foxes use a wide variety of features for den and rest sites [13] so these are unlikely to be density- limiting factors. Increases in the amount and suitability of suburban habitats, combined with increases in wildlife feeding by householders [8] may have supported population growth once colonisation occurred. There has also been a move away from lethal control and foxes have adapted their behaviour to reduce the risk of road mortality, although road traffic accidents remain a common cause of death [45]. Social group density is therefore likely to be affected by inter-group competition for resources, and overall density driven by social group size, which is itself limited by intra-group competition.

We found no relationship between urban fox group densities and landscape composition. Furthermore, densities were very similar across sites in Brighton despite these areas contrasting in housing density and sociological composition. This indicates that previous studies using sociological data to predict fox densities [36] are no longer likely to be valid in modern suburban areas. Anthropogenic food sources, which can make up a considerable proportion of urban fox diet [46], have changed in availability, with increases in the provision by residents [8, 47]. These resources are typically provided in residential gardens, and their presence may reduce the influence of natural resources on fox density. The dynamics of such potential inter-relationships and associated behavioural mechanisms are unknown, and merit further study.

The documented spread in distribution and potential increase in abundance of foxes in urban areas has also been reported in Europe (e.g., [48]) and globally (e.g., [12]). It is still unclear why this phenomenon is occurring but suggestions include an increase in availability of habitats and resources that foxes can behaviourally adapt to and exploit [48], including suburban areas, which are expanding to provide housing for the growing human population.

Mean density of badger main setts has been estimated nationally for different rural land use classes in England and Wales to give an overall density estimate of 0.485km^-2^ [34]. Our badger estimate from Brighton is five times greater than this, consistent with Davison et al (2008) who assert that urban sett counts are typically higher than rural ones. However, our replicates showed low precision, again in agreement with Davison et al. (2008). The habitat characteristics required by badgers (cover and sloping terrain of a suitable substrate for digging) are patchily distributed in urban areas hence urban badgers occupy non-contiguous home ranges and occur at variable social group densities [35]. Wider extrapolations for urban land classes are therefore untenable without further extensive study across multiple cities. Our density estimate of 2.41 km^-2^ is not as high as the 4.30 km^-2^ previously determined for one area of the same city (Brighton), but approximates to estimates from other urban areas (Hastings 2.05 km^-2^ and Bristol 1.88 km^-2^) (data from [35]). Further urban badger social group density estimation is required to correlate urban landscape structure with badger population size. Our method gives a viable approach to determining badger group density within suburban habitats where data are currently sparse.

Our method allows estimation of relative fox group densities within and between cities, as validated by similar results from intensive surveys at one site. However, further calibration against a baseline estimate is recommended, at a range of different sites and cities. As the home range size and territorial spacing of both species varies a sensitivity analysis of the integration method is recommended; i.e. calculation of robustness at different densities. The home range estimates in Brighton (see [39]) are small compared to previous studies, and ranges may be larger elsewhere, with fox cubs potentially moving between den locations that are greater than 200m apart. In such a scenario, the 200m integration method would lead to double counting and over-estimation of density. Fox social groups may have multiple litters in one year, which, if fragmented to smaller groups early, may also result in over-estimation. Likewise, badger group inter-sett distances might be less than, or exceed, the 150m spacing used here. Therefore, sensitivity analyses are also required for integration distance.

The resident response rate of 19% provided sufficient records of both fox and badger sightings to allow aggregation of sightings into centroids within GIS analysis, as representative of social groups. All sites provided sufficient data for estimates, demonstrating the ability to utilise records from residents across a country to determine numbers of social groups of both urban carnivores. However, response rate was variable, with questionnaire recipients from some sites being unresponsive. A low response rate in an area of low carnivore density would be at risk of insufficient returns, therefore incentives for response might be necessary. Responses via one route only (e.g. postal returns) should be monitored and a secondary route (e.g. door to door) followed up if response rate is low. Efficiency may also be increased by tailoring delivery effort to housing density such that the same effort per unit area is deployed across all areas regardless of number of dwellings therein.

The method presented here could be adapted to compare densities in different cities with residential suburban areas across the globe and be used as a baseline to study spatiotemporal changes in fox and badger densities where this is desired. For example, foxes are colonising cities in other parts of Europe where there are concerns of zoonotic disease transmission, including rabies and *Echinococcus spp*. [49, 50] and simple, efficient, and low-cost assessment of densities is paramount. Survey approaches are determined by ecological characteristics of the focal species, including social unit and group size, spatial distribution and space use, territoriality and territory size. Table 3 shows a summary of key ecological traits of selected urban carnivores [2, 39, 51, 52]. To enable application of the method to other urban carnivores, such traits require consideration and the method adapted accordingly. Exclusive territories are essential for the method hence it is appropriate for coyotes *(Canis latrans)*, kit foxes (*Vulpes macrotis*) and stone martens (*Martes foina*), but not for solitary species with overlapping territories, such as skunks (*Mephitis spp*.) and raccoons (*Procyon lotor*). The clustering unit can be determined either by central den locations or litter locations, and mean territory size is required for buffer distance selection. For example, to estimate group density for coyotes, a large area would need to be surveyed due to extensive ranging. In this case a scatter approach might be engaged such that questionnaires cover a greater area with little or no additional effort. Ideally, the method would be employed in multi-species assessments, for example where kit foxes, coyotes and red foxes are sympatric (e.g. [53]) to maximise efficiency. Our finding of increased fox social group density relative to previous estimates has implications for ongoing disease surveillance and contingency plans, in particular relating to controlling potential outbreaks of rabies and HAE. However, foxes are also considered potentially highly important in suppressing Lyme disease (*Borrelia sp*.*)* hosts in areas of human habitation [54], hence higher fox densities may be beneficial in controlling some zoonotic diseases.

**Table 3:** Ecological traits of selected urban carnivores.

	Red fox(*Vulpes vulpes*)	Coyote(*Canis latrans*)	Kit fox(*Vulpes macrotis*)	European badger(*Meles meles*)	Stone marten(*Martes foina*)^c^	Striped skunk(*Mephitis spp*.*)*	Raccoon(*Procyon lotor*)	Bobcat (*Lynx rufus*)
Distribution	Northern hemisphere, Australia	North America	SW USA, NW Mexico	Europe, Asia	Europe, Central Asia	North America	North America	North America
Social group	Yes	Yes	Yes	Yes	No	No	Males	No
Urban social group size	2.2–6.6	4–6	1–3	5.5	Solitary	Solitary	-	Solitary
Adult urban density	2–12 km^-2^	0.3–3 km^-2^	-	33 km^-2 **b**^	4.7–5.8 km^-2^	2–7 km^-2^	125–333 km^-2^	0.04–0.28 km^-2^
Exclusive territories	Yes	Yes	Yes	Yes	Yes—Intersexual	No	No	No
Size of urban home range	0.14 km^2 **a**^	3–36 km^2^	1.2 km^2^	0.09 km^2^ (group)	1.13 (m)0.37 (f) km^2^	0.51–0.64 km^2^	0.05–0.79 km^2^	1.3–6.4 km^2^
Urban extent in home range	Fully	Mixed	Fully	Fully	Fully	Fully	Fully	Fully
Seasonal breeder	Yes	Yes	Yes	Yes	Yes	Yes	Yes	Yes
Typically one litter per social group	Yes	Yes	Yes	Yes	No	No	No	No

A summary of ecological traits of selected urban carnivores. Information summarised from [2] with data from ^a^ [39], ^b^ [52] and ^c^ [53]; (-) denotes limited information.
